# Long-term effects of vertical bone augmentation: a systematic review

**DOI:** 10.1590/1678-775720150357

**Published:** 2016

**Authors:** Johan Anton Jochum Keestra, Obada Barry, Lianne de Jong, Gerhard Wahl

**Affiliations:** 1 - Ordentall, Rotterdam, Netherlands.; 2 - Praktijk voor Parodontologie en Implantologie, Tilburg, Netherlands.; 3 - Universität Bonn, Poliklinik für Chirurgische Zahn-, Mund- und Kieferheilkunde, Bonn, Germany.; 4 - Dental Clinics Zuiderterras, Rotterdam, Netherlands.

**Keywords:** Alveolar ridge augmentation, Dental implants, Atrophy, Alveolar bone loss, Bone substitutes

## Abstract

Extraction, periodontitis, or trauma can cause a reduction on the alveolar ridge. This could result in an insufficient alveolar bone width and height. Different techniques of vertical bone augmentation are described in literature. However, nowadays there is not enough evidence against lateral augmentation procedures to verify if these techniques are stable over a long period of time.

**Objective:**

This review analyses the different techniques that are used to vertically augment the bone and evaluate if these techniques are stable over a long period of time.

**Material and Methods:**

The MEDLINE-PubMed database was searched from its earliest records until December 22, 2014. The following search term was used: Alveolar Ridge augmentation [MESH]. Several journals were hand searched and some authors were contacted for additional information. The primary outcome measure that was analyzed was marginal bone level change around dental implants in the augmented sites, and the secondary outcomes were survival and success rates of dental implants placed in the augmented sites.

**Results:**

The search yielded 203 abstracts. Ultimately, 90 articles were selected, describing 51 studies meeting the eligibility criteria. The marginal bone level change for the inlay technique and vertical guided bone regeneration are in agreement with the success criteria. Alveolar distraction showed more marginal bone level change after the first year of loading, and for the inlay technique very few studies were available.

**Conclusions:**

Based on the available data in the current existing studies with a follow-up period of at least 4 to 5 years, one can summarize that there seems to be a trend that the onlay technique, alveolar distraction, and vertical guided bone regeneration are stable for at least 4 to 5 years.

## INTRODUCTION

Since Brånemark introduced a new dental treatment, a machined titanium implant, a new treatment option became available^3^. If there is sufficient bone quantity and quality, a dental implant could be a predictable treatment option. In literature, a survival rate over 95% in non-compromised patients is reported^32^. Therefore, dental implants have become a reliable treatment option for patients missing one or multiple teeth. However, unfavourable conditions of the alveolar bone due to periodontitis, extraction, or trauma provoke decrease in the alveolar ridge due to bone atrophy. Such bone atrophy could cause challenging interarch relationship in vertical, transverse, and sagittal planes, which may cause incorrect dental implant placement from a functional and aesthetic point of view^19^.

To provide adequate bone volume and to assure an adequate aesthetic result, bone augmentation procedures are sometimes a prerequisite for successful dental implant treatment. There are different techniques to augment the bone, such as:

1. Onlay grafting. The graft material will be placed on top of the defect to increase height or width of the alveolar bone. The graft is immobilised with dental implants, screws, or plates^52^.
2. Inlay grafting. A part of the alveolar ridge is surgically separated and a graft material is placed between the two sections^52^.
3. Ridge expansion. A part of the alveolar ridge is longitudinally split to widen the ridge and allow placement of a graft, an oral implant, or both^35^.
4. Distraction osteogenesis. A gradual, controlled displacement of a surgically prepared fracture. The two bone fragments are slowly pulled apart, and new bone will arise in the gap ^26^.
5. Guided bone regeneration (GBR). A space is maintained by a barrier membrane, which will be filled with new bone^67^.

Different materials can be used for augmentation:

1. Autogenous bone graft. This bone graft is taken from the same patient in an adjacent or remote site. This material is considered to be the “gold standard”, while it is biologically compatible and provides a scaffold for new bone formation^77^.
2. Allograft. This bone graft is harvested from human cadavers and processed by methods such as freezing or demineralising and freezing^67^.
3. Xenograft. This is a graft material derived from animals, usually bovine bone. It is processed to completely remove the organic component^13^.
4. Alloplastic graft. This bone graft is a synthetic bone substitute made up of bioactive glass or calcium phosphates^112^.
5. Osteoinductive material. This material stimulates the osteoprogenitor cells to differentiate into osteoblasts and accelerate new bone formation. The most common are bone morphogenetic proteins (BMPs), platelet rich plasma (PRP), and leukocyte platelet rich fibrin (L-PRF)^33^.

Each type of augmentation material may be used combined with a variety of different surgical techniques.

The rationale for the use of a vertical bone augmentation is to improve the vertical dimension of the bone. If the use of a vertical bone augmentation technique is needed, the clinician needs to decide which technique and which material should be used to vertically augment the bone. When the vertical bone augmentation is successful, one can proceed for dental implant placement. The aim of this review is to analyze the success, survival rates of dental implants, and the marginal bone level change around dental implants placed in the augmented area. Marginal bone level change is most often controlled through x-rays in the maintenance phase to demonstrate and secure implant success.

## MATERIAL AND METHODS

The following analysis was performed in a different way according to the guidelines of the Cochrane Collaboration and the principles of the PRISMA (Preferred Reporting Items for Systemic Reviews and Meta-Analyses) statement for a systematic review^46^ ^,^ ^69^.

### Focused question (PICO)

We focused on the following question: “Do vertical bone augmentation have a long-term predictable stability?”.

### Search strategy

The MEDLINE-PubMed database was searched from its earliest records until December 22, 2014. The following search term was used: Alveolar Ridge augmentation [MESH]. In addition, a manual search was carried out concerning issues from the past 10 years of the following journals: Clinical Implant Dentistry and Related Research, Clinical Oral Implants Research, European Journal of Oral Implantology, Implant Dentistry, International Journal of Oral and Maxillofacial Implants, International Journal of Oral and Maxillofacial Surgery, Journal of Oral Implantology, Journal of Oral and Maxillofacial Surgery, Journal of Clinical Periodontology, Journal of Periodontal Research, and the Journal of Periodontology.

### Study inclusion and exclusion criteria

The selection process was performed by two masked reviewers (OB and JK). The studies were analyzed according to the following inclusion criteria:

1. All studies in which at least 10 patients were treated and had a follow-up of at least 12 months.
2. Patients presenting deficient edentulous ridges caused by atrophy, periodontal disease, and trauma were considered.
3. The following surgical procedures were considered: onlay bone grafts, split-ridge/ridge expansion techniques/inlay technique (vertical direction), alveolar distraction osteogenesis, and guided bone regeneration procedures.
4. Articles related to dental implants were considered for inclusion.
5. No specific dental implant system was excluded.
6. No specific augmentation material was excluded.
7. Only studies in the English language were included.

The following exclusion criteria were used:

1. Patients with bone defects caused by congenital malformations, after ablation of tumors, or osteoradionecrosis.
2. The following surgical procedures were excluded: sinus floor elevation by a lateral approach, Le Fort I osteotomy with interpositional grafts, revascularized free flaps, socket preservation techniques, and correction of dehiscences and fenestrations.
3. Duplicated studies.

### Outcome variables

The primary outcome was: marginal bone level change around dental implants in the augmented sites. The following recall moments were noted: baseline (placement of the final crown, start loading), year 1, 2, 3, 4, and 5 of loading. The secondary outcomes were survival and success rates of dental implants placed in the augmented sites. Implant survival was evaluated using Simonis, et al.^97^ (2010), being implant removal the survival criterion. Implant success was evaluated using Albrektsson, et al.^5^ (2012), and the success criteria were absence of persistent pain or dysesthesia, absence of peri-implant infection with suppuration, absence of mobility, absence of continuous peri-implant radiolucency, less than 1.5 mm of peri-implant bone resorption during the first year of function, and less than 0.2 mm in subsequent years.

### Data extraction

The title and abstract of studies with potential relevance for the review were obtained and screened independently by two masked reviewers (OB and JK). Studies without abstract, but with a title suggesting relevance to the subject of the review, were selected for full text screening. The selected full-text articles were independently read in detail to verify whether they passed the inclusion/exclusion criteria. The references of the full text articles were screened for any relevant additional articles. Studies that fulfilled all the selection criteria were processed for data extraction. Discrepancies regarding the inclusion or exclusion of studies were resolved by discussion between the reviewers (OB and JK). The extracted data included: year of publication, design of the study, number of patients *per* study arm, defect type, surgical procedure, donor site, number of dental implants, timing of implants, follow-up time, primary outcome measure at baseline (placement of the final crown, start loading), year 1, 2, 3, 4, and 5 of loading, and secondary outcomes measures. The quality of the various studies were not considered in the final analysis, therefore, no quality assessment has been done.

### Statistical analyses

Data of the included studies were extracted and inserted into a database. Mean values and standard deviations were extracted from the data. If no standard deviation was available, it was recalculated by the formula (SE=SD/√n) where *n* is the sample size. When mean follow-up period was used, it was recalculated, if possible, for every year; if not, the nearest full year was used. If there were insufficient data available, the corresponding authors were contacted for additional data. The available data were recalculated in order to present the data like marginal bone level change at baseline (placement of the final crown, start loading), year 1, 2, 3, 4, and 5 of loading, and the latest available data for survival and success rates were noted. The data of this review was statistically analyzed using the program SPSS 21.0 (IBM Corp. Released 2012. IBM SPSS Statistics for Windows, Version 21.0. Armonk, NY: IBM Corp.).

## RESULTS

**Figure 1:**
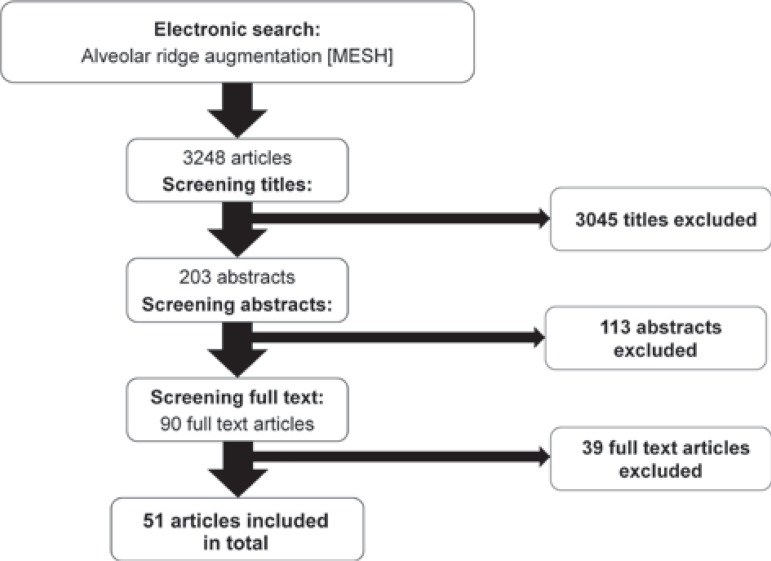
Search strategy

**Figure 2:**
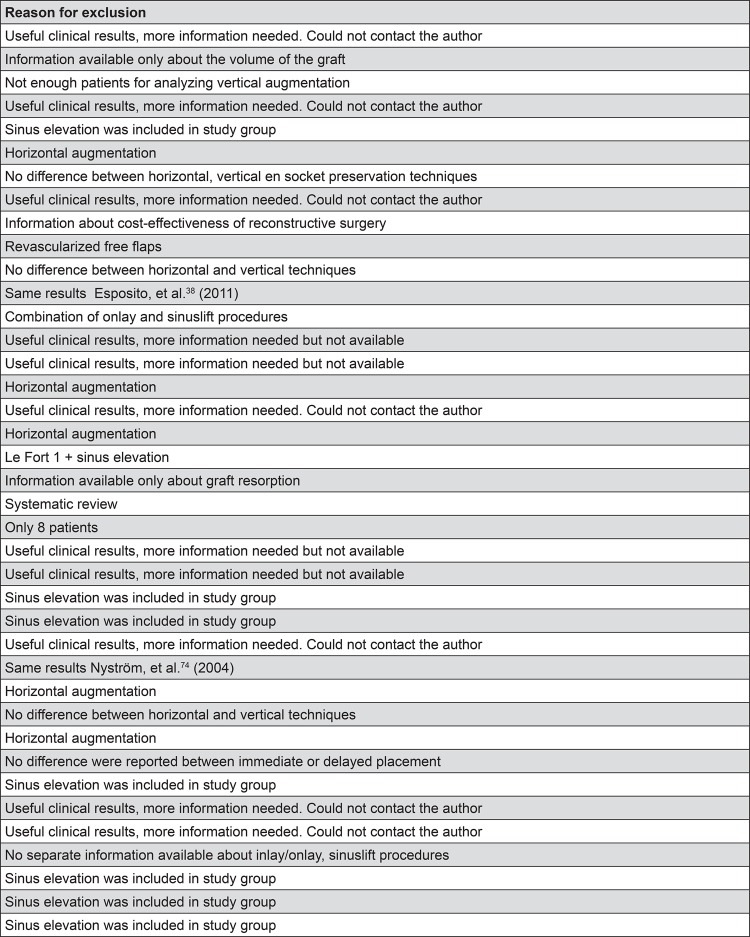
Characteristics of the 39 studies excluded

The initial search resulted in a total of 3248 articles (Figure 1). After screening the titles, 203 abstracts were included for further analysis. Analysis of the abstracts resulted in 90 potential articles. In the third phase, the full-text articles of the remaining 90 articles were evaluated, of which 39 articles^2^ ^,^ ^8^ ^,^ ^9^ ^,^ ^12^ ^,^ ^14^ ^,^ ^18^ ^,^ ^23^ ^,^ ^24^ ^,^ ^27^ ^,^ ^28^ ^,^ ^30^ ^,^ ^39^ ^,^ ^41^ ^,^ ^44^ ^,^ ^45^ ^,^ ^48^ ^,^ ^49^ ^,^ ^51^ ^,^ ^56^ ^,^ ^59^ ^,^ ^60^ ^,^ ^62^ ^,^ ^64^ ^,^ ^68^ ^,^ ^73^ ^,^ ^75^ ^,^ ^84^ ^,^ ^89^ ^,^ ^90^ ^,^ ^92^ ^-^ ^94^ ^,^ ^98^ ^,^ ^102^ ^,^ ^103^ ^,^ ^107^ ^,^ ^108^ ^,^ ^111^ ^,^ ^113^ did not pass the inclusion criteria (Figure 2). A screening of the reference lists of the full text articles did not result in any additional articles. In Table 1, the main characteristics of the 51 included studies are summarized^1^ ^,^ ^6^ ^,^ ^7^ ^,^ ^10^ ^,^ ^15^ ^,^ ^16^ ^,^ ^20^ ^-^ ^22^ ^,^ ^25^ ^,^ ^29^ ^,^ ^31^ ^,^ ^34^ ^,^ ^36^ ^-^ ^38^ ^,^ ^40^ ^,^ ^42^ ^,^ ^43^ ^,^ ^50^ ^,^ ^53^ ^,^ ^55^ ^,^ ^57^ ^,^ ^58^ ^,^ ^61^ ^,^ ^63^ ^,^ ^65^ ^,^ ^66^ ^,^ ^70^ ^-^ ^72^ ^,^ ^74^ ^,^ ^78^ ^-^ ^86^ ^,^ ^91^ ^,^ ^95^ ^,^ ^96^ ^,^ ^99^ ^,^ ^100^ ^,^ ^104^ ^-^ ^106^ ^,^ ^109^ ^,^ ^110^ ^,^ ^114^. Only the treatment groups of interest are represented. For vertical bone augmentation, four different techniques were used and the results will be presented separately. In Table 2, the characteristics of the different vertical augmentation techniques are presented.

**Table 1:** Characteristics of the 51 studies included

Reference	Study design	Number of patients	Defect type (type of atrophy)	Surgical procedure	Donor materials	Number of implants	Timing of implants	Follow-up	Implant survival (%)	Implant success (%)
Kim, et al.^57^ (2013)	Retrospective study	14 28	Max + Man + Hor + Ver Max + Man + Hor + Ver	Alveolar distraction Onlay technique	Autogenous (Ramus)	41 61	Del 4.9 months Del 6.2 months	7.1 ± 1.7 years 8.2 ± 2.0 years	97.3 94.1	92.7 90.2
Pérez-Sayáns, et al.^79^ (2013)	Retrospective study	14	Max + Man + Hor + Ver	Alveolar distraction		50	Del 3.0 months	3 years	100	96
Korpi, et al.^58^ (2012)	Prospective study	22	Man + Hor + Ver	Onlay technique	Autogenous (Iliac)	48	Imm	3-9 years	100	96
De Riu, et al.^31^ (2012)	Prospective study	15	Man + Hor + Ver	Onlay technique	Autogenous (Coronoid)	40	Del 6.0 months	2 years	95	96.7
Zwetyenga, et al.^114^ (2012)	Retrospective study	37	Man + Hor + Ver	Alveolar distraction		127	Del 5.8 months	5.2 years	100	96.2
Sezer, et al.^95^ (2012)	Prospective study	10	Man + Hor + Ver	Alveolar distraction		40	Del 4.0 months	3 years	100	100
Kawakami, et al.^53^ (2013)	Controlled split mouth study	12	Man + Hor + Ver Man + Hor + Ver	Inlay technique Inlay technique	Autogenous (Ramus) Alloplastic graft	22 22	Del 6.0 months	1 year	95.5 95.5	90.9 90.9
Annibali, et al.^6^ (2012)	Retrospective study	5	Man + Hor + Ver	Vertical guided bone regeneration	Autogenous (Ramus) + Allograft	16	Imm	1.0 ± 0.1 years	100	81.3
Nissan, et al.^72^ (2012)	Prospective study	40	Max + Hor + Ver	Onlay technique	Allograft	83	Del 6.0 months	4.0 ± 1.8 years	98.8	X
Esposito, et al.^38^ (2012)	Randomized control trial	30	Man + Hor + Ver	Inlay technique	Xenograft	61	Del 5.0 months	3 years	100	X
Chiapasco, et al.^20^ (2012)	Prospective study	11 7	Max + Man + Hor + Ver	Onlay technique	Autogenous (Ramus) Autogenous (Calvarium)	29 31	Del 4-5 months Del 6-7 months	1.6 years	100 100	93.1 90.3
Acocella, et al.^1^ (2012)	Prospective study	16	Max + Hor + Ver	Onlay technique	Allograft	34	Del 6.0 months	1.5-2.5 years	100	X
Ludovichette, et al.^65^ (2011)	Prospective study	19	Max + Man + Hor + Ver	Onlay technique	Alloplastic graft	49	Imm	3 years	100	100
Rigo, et al.^85^ (2011)	Retrospective study	17	Max + Man + Hor + Ver	Inlay/onlay technique	Allograft	60	Del 6.0 months	2.2 years	100	100
Canullo, et al.^15^ (2010)	Prospective study	20	Max + Man + Hor + Ver	Onlay technique	Alloplastic graft	42	Imm	2 years	100	100
Todisco, et al.^100^ (2010)	Prospective study	20	Max + Man + Hor + Ver	Vertical guided bone regeneration	Allograft	64	Del 12 months	1.2 years	100	97
Corinaldesi, et al.^29^ (2009)	Retrospective study	24	Max + Man + Hor + Ver	Onlay technique	Autogenous (Ramus)	56	Imm / Del 8-9 months	3-8 years	100	96.4
Le, et al.^61^ (2010)	Prospective study	15	Max + Man + Hor + Ver	Vertical guided bone regeneration	Allograft	32	Del 4-5 months	1.4 years	100	100
Pelo, et al.^78^ (2010)	Prospective study	19	Man + Hor + Ver	Onlay technique	Autogenous (Ramus)	141	Del 4.0 months	4.0 years	96	91
Sbordone, et al.^91^ (2009)	Retrospective study	40	Max + Man + Hor + Ver	Onlay technique	Autogenous (Ramus, Iliac)	109	Del 3-5 months	3.0 years	99.1	X
Elo, et al.^36^ (2009)	Retrospective study	65 17	Max + Man + Hor + Ver	Onlay technique Alveolar distraction	Autogenous (Iliac, Chin, Retromolar, Tibia)	184 56	Del 4-5 months	3-5.1 years	98.4 98.2	96.7 98.2
Ettl, et al.^40^ (2010)	Retrospective study	30	Max + Man + Hor + Ver	Alveolar distraction		82	Del 4.5 months	4.2 years	95.1	X
Nissan, et al.^71^ (2011)	Prospective study	31	Max + Hor + Ver	Onlay technique	Allograft	63	Del 6.0 months	2.8 ± 1.3 years	98.1	X
Felice, et al.^42^ (2009)	Prospective study	10 10	Man + Hor + Ver	Inlay technique Onlay technique	Autogenous (Iliac)	20 23	Del 3-4 months	1.5 years	100 100	90 86.9
Nissan, et al.^70^ (2011)	Prospective study	21	Man + Hor + Ver	Onlay technique	Allograft	85	Del 6.0 months	3.1 ± 1.4 years	95.1	X
Urban, et al.^105^ (2009)	Retrospective study	28	Max + Man + Hor + Ver	Vertical guided bone regeneration	Autogenous (Ramus, Chin)	54	Del 6-9 months	2.8 years	100	94.7
Carinci, et al.^16^ (2009)	Retrospective study	21	Man + Hor + Ver	Onlay technique	Allograft	63	Del 6.0 months	1.7 years	96.8	X
Robiony, et al.^86^ (2008)	Prospective study	12	Man + Hor + Ver	Alveolar distraction + Inlay technique	Autogenous (Iliac)	47	Del 6.0 months	5 years	97.9	91.5
Pieri, et al.^80^ (2008)	Prospective study	16	Max + Man + Hor + Ver	Vertical guided bone regeneration	Autogenous (Ramus) +Xenograft	44	Del 8-9 months	2 years	100	93.1
Bianchi, et al.^10^ (2008)	Prospective study	5 6	Man + Hor + Ver	Inlay technique Alveolar distraction	Autogenous (Iliac)	21 16	Del 3-4 months Del 4-5 months	1.8 years 2.5 years	100 100	95.2 93.7
Chiapasco, et al.^25^ (2007)	Prospective study	8 9	Man + Hor + Ver	Onlay technique Alveolar distraction	Autogenous (Ramus)	19 21	Del 4-5 months Del 3 months	2-4 years	100 100	89.5 94.7
Uckan, et al.^104^ (2007)	Retrospective study	21	Max + Man + Hor + Ver	Alveolar distraction		42	Del 3-4 month	2.7 years	88	X
Polo, et al.^81^ (2007)	Prospective study	10	Man + Hor + Ver	Alveolar distraction		34	Del 3-4 months	1.0 ± 0.3 years	100	X
Levin, et al.^63^ (2007)	Retrospective study	50	Max + Man + Hor + Ver	Onlay technique	Autogenous (Ramus, Iliac)	129	Del 4-6 months	2.0 ± 0.9 years	96.9	91.9
Smolka, et al.^99^ (2006)	Prospective study	10	Man + Hor + Ver	Onlay technique	Autogenous (Calvarium)	20	Del 6.0 months	2.5 years	95	X
Enislidis, et al.^37^ (2005)	Retrospective study	32	Man + Hor + Ver	Alveolar distraction		94	Del 3-5 months	3.0 years	95.7	X
van der Meij, et al.^106^ (2005)	Retrospective study	17	Man + Hor + Ver	Onlay technique	Autogenous (Calvarium)	34	Imm	4.3 years	88.2	88.2
Nyström, et al.^74^ (2004)	Retrospective study	30	Max + Hor + Ver	Onlay technique	Autogenous (Iliac)	177	Imm	10 years	72,8	X
Chiapasco, et al.^21^ (2004)	Prospective study	37	Max + Man + Hor + Ver	Alveolar distraction		138	Del 3 months	2.8 years	100	94.2
Chiapasco, et al.^22^ (2004)	Prospective study	5 10	Max + Man + Hor + Ver	Vertical guided bone regeneration Alveolar distraction	Autogenous (Ramus)	12 34	Del 6-7 months Del 3-4 months	1-3 years	100 100	75 94.1
Raghoebar, et al.^83^ (2002)	Prospective study	10	Man + Hor + Ver	Alveolar distraction		20	Del 2-3 months	0.9 years	95	X
Jensen, et al.^50^ (2002)	Prospective study	28	Max + Man + Hor + Ver	Alveolar distraction		84	Del 3-4 months	1-4.4 years	90.4	X
Rachmiel, et al.^82^ (2001)	Retrospective study	14	Max + Man + Hor + Ver	Alveolar distraction		23	Del 2-3 months	0.5-1.7 years	95.7	X
Simion, et al.^96^ (2001)	Retrospective study	6 11 32	Max + Man + Hor + Ver	Vertical guided bone regeneration	Allograft Autogenous (Ramus, Chin)	17 26 82	Imm	5.3 years 3.3 years 2.5 years	94.1 100 100	94.1 96.1 100
Gaggl, et al.^43^ (2000)	Prospective study	34	Max + Man + Hor + Ver	Alveolar distraction		62	Imm	1 year	96	X
Keller, et al.^55^ (1999)	Retrospective study	28 4	Max + Hor + Ver	Onlay technique	Autogenous (Iliac)	183 21	Imm Del 4-6 months	5.6 years	86.3 91	X
Verhoeven, et al.^109^ (1997)	Prospective study	13	Man + Hor + Ver	Onlay technique	Autogenous (Iliac)	72	Imm	2.4 ± 0.9 years	100	X
McGrath, et al.^66^ (1996)	Retrospective study	18	Man + Hor + Ver	Onlay technique	Autogenous (Iliac)	36	Imm	1.4 years	91.6	91.6
Vermeeren, et al.^110^ (1996)	Retrospective study	31	Man + Hor + Ver	Onlay technique	Autogenous (Iliac)	78	Imm	5 years	89.7	X
Astrand, et al.^7^ (1996)	Retrospective study	17	Max + Hor + Ver	Onlay technique	Autogenous (Iliac)	92	Imm	3-5 y	75	X
Donovan et al.^34^ (1994)	Retrospective study	24	Max + Man + Hor + Ver	Onlay technique	Autogenous (Calvarium)	43 50	Imm Del 6-8 months	1.5 years 2.6 years	97.7 86	X X

**Table 2:** Characteristics of the different vertical augmentation techniques

	Alveolar distraction	Inlay technique	Onlay technique	Vertical guided bone regeneration
Patients (n)	345	74	700	138
Implants (n)	1011	206	2155	347
Survival rate (%)	97.1	98.5	94.7	99.3
Success rate (%)	95.5	93.4	93.2	90.7

### Alveolar distraction (Table 1, Figure 3)

The 51 included articles provided 17 studies^10^ ^,^ ^21^ ^,^ ^22^ ^,^ ^25^ ^,^ ^36^ ^,^ ^37^ ^,^ ^40^ ^,^ ^43^ ^,^ ^50^ ^,^ ^57^ ^,^ ^79^ ^,^ ^81^ ^-^ ^83^ ^,^ ^95^ ^,^ ^104^ ^,^ ^114^ with alveolar distraction, and one study^86^ used a combination of the inlay technique and alveolar distraction. Eight studies were retrospective while 10 were prospective. A total of 333 patients with a vertical resorption of partially or totally edentulous alveolar ridges were treated with intraoral intraosseous or extraosseous devices. Twelve patients were treated with a combination of inlay technique and vertical distraction. In total, 1011 dental implants were placed after 3 to 6 months, and the mean was 3.8 months after the completion of the distraction. After the start of loading, the follow-up ranged from 1 to 7.1 years and the mean was 2.9 years. The survival rates for the dental implants in alveolar distracted bone ranged from 88 to 100% and the mean was 97.1%. Unfortunately, only nine studies evaluated the implant success rate. This ranged from 92.7 to 100.0%, and the mean was 95.5%.

**Figure 3:**
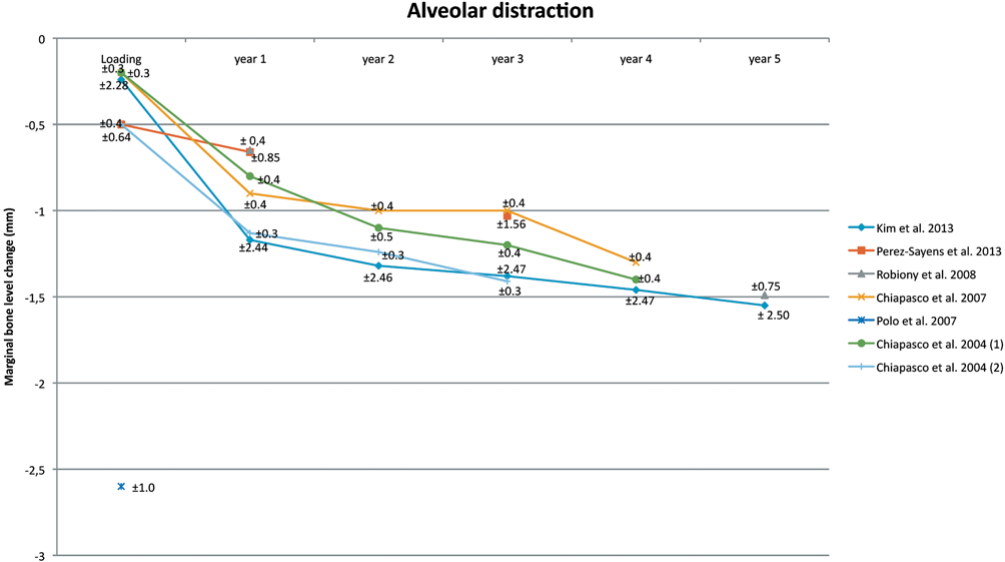
Alveolar distraction. Mean and Standard Deviation are indicated

Only seven studies^21^ ^,^ ^22^ ^,^ ^25^ ^,^ ^57^ ^,^ ^79^ ^,^ ^81^ ^,^ ^86^ out of the 17 which used alveolar distraction as a treatment presented the marginal bone level change in their results. The marginal bone level change is shown in Figure 3. Only four studies presented the results for a follow-up period of 4 or 5 years. At baseline, the marginal bone level change is around -0.20 – -0.50 mm, 1^st^ year of loading -0.65 – -1.17 mm, 2^nd^ year of loading -1.00 – -1.32 mm, 3^rd^ year of loading -1.00 – -1.41 mm, 4^th^ year of loading -1.30 – -1.46 mm, and 5^th^ year of loading -1.49 – 1.55 mm.

### Inlay technique (Table 1, Figure 4)

The 51 articles included provided four studies^10^ ^,^ ^38^ ^,^ ^42^ ^,^ ^53^ with inlay technique, and one study^85^ used a combination of onlay and inlay techniques. Of these, two were prospective studies; one, a a split mouth study; and one, a randomized clinical trial. A total of 57 patients with a vertical resorption of partially or totally edentulous alveolar ridges were treated with the inlay technique. Seventeen patients were treated with a combination of onlay and inlay techniques. Three different donor materials for the bone where used: autogenous (iliac^10^ ^,^ ^42^, ramus^53^), xenografts^38^, and alloplastic grafts^53^. In total, 206 dental implants were placed after 3 to 6 months, and the mean was 4.6 months after the healing of the inlay technique. After the start of loading, the follow-up ranged from 1 to 3 years, and the mean was 1.7 years. Survival rates for the dental implants in bone from the inlay technique ranged from 95.9 to 100.0%, and the mean was 98.5%. Unfortunately, only four studies evaluated the implant success rate, which ranged from 90.9 to 100.0%, and the mean was 93.4%.

**Figure 4:**
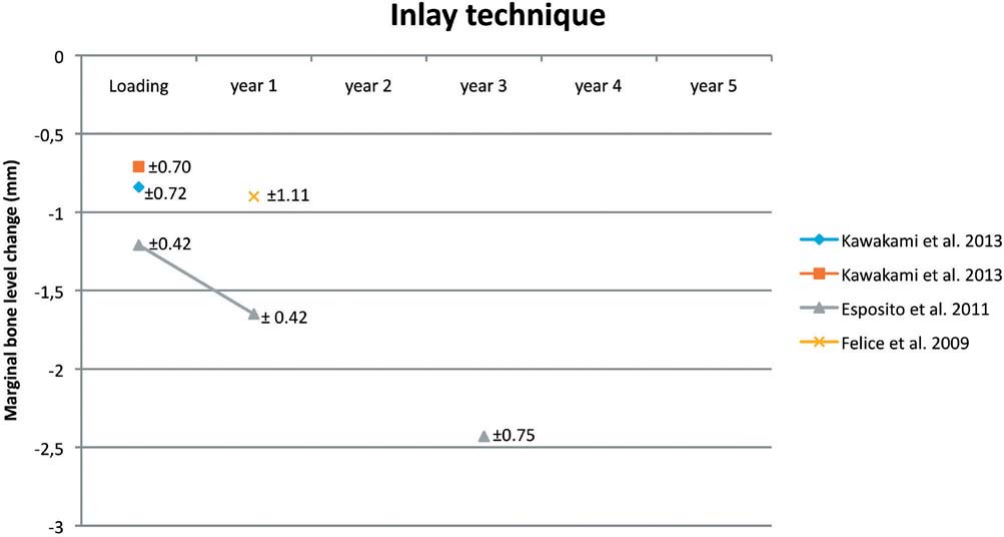
Inlay technique. Mean and Standard Deviation are indicated

Only three studies^38^ ^,^ ^42^ ^,^ ^53^ out of the four which used the inlay technique presented the marginal bone level change in their results. The marginal bone level change is shown in Figure 4. One study^53^ has different treatment groups, therefore, it is shown twice in the figure. None of the studies showed a long-term follow-up. At baseline, the marginal bone level change is around -0.71 – -1.21 mm, 1^st^ year of loading -0.90 – -1.65 mm, and 3^rd^ year of loading -2.43 mm.

### Onlay technique (Table 1, Figure 5)

The 51 articles included provided 27 studies^1^ ^,^ ^7^ ^,^ ^15^ ^,^ ^16^ ^,^ ^20^ ^,^ ^25^ ^,^ ^29^ ^,^ ^33^ ^,^ ^35^ ^,^ ^41^ ^,^ ^54^ ^,^ ^56^ ^,^ ^57^ ^,^ ^62^ ^,^ ^64^ ^,^ ^65^ ^,^ ^69^ ^-^ ^71^ ^,^ ^73^ ^,^ ^77^ ^,^ ^85^ ^,^ ^91^ ^,^ ^99^ ^,^ ^106^ ^,^ ^109^ ^,^ ^110^ with onlay technique, and one study^84^ used a combination of inlay and onlay techniques. Thirteen studies were retrospective while 14 were prospective. A total of 683 patients with a vertical resorption of partially or totally edentulous alveolar ridges were treated with the onlay technique. Seventeen patients were treated with a combination of onlay and inlay techniques. Three different donor materials for the bone where used: autogenous (iliac^7^ ^,^ ^36^ ^,^ ^42^ ^,^ ^55^ ^,^ ^58^ ^,^ ^63^ ^,^ ^66^ ^,^ ^74^ ^,^ ^91^ ^,^ ^109^ ^,^ ^110^, ramus^20^ ^,^ ^25^ ^,^ ^29^ ^,^ ^36^ ^,^ ^57^ ^,^ ^78^, calvarium^20^ ^,^ ^34^ ^,^ ^99^ ^,^ ^106^, chin^36^, tibia^36^, and coronoid^31^), allografts^1^ ^,^ ^16^ ^,^ ^70^ ^-^ ^72^ ^,^ ^85^, and alloplastic grafts^15^. In total, 910 dental implants were placed immediately, 1245 dental implants were placed after 3 to 9 months, and the mean was 5.5 months after the healing of the onlay technique. After the start of loading, the follow-up ranged from 1.4 to 10 years, and the mean was 3.5 years. Survival rates for the dental implants in bone from the onlay technique ranged from 72.8 to 100.0%, and the mean was 94.7%. Unfortunately, only 14 studies evaluated the implant success rate, which ranged from 86.9 to 100.0%, and the mean was 93.2%.

**Figure 5:**
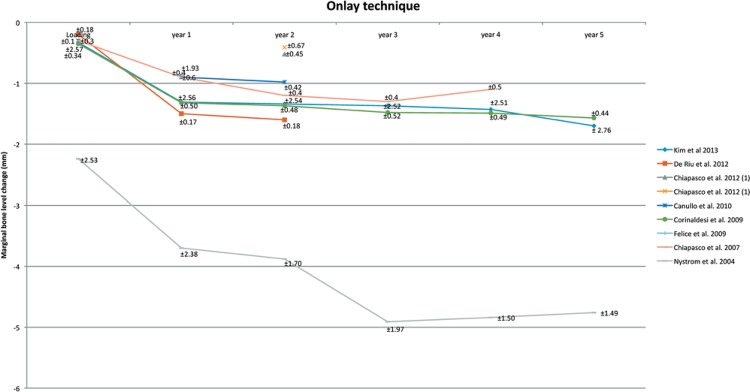
Onlay technique. Mean and Standard Deviation are indicated

Only eight studies^15^ ^,^ ^20^ ^,^ ^25^ ^,^ ^29^ ^,^ ^31^ ^,^ ^42^ ^,^ ^57^ ^,^ ^74^ out of the 27 which used the onlay technique as a treatment presented the marginal bone level change in their results. The marginal bone level change is shown in Figure 5. One study^20^ has different treatment groups, therefore, it is shown twice in the figure. Only four studies presented the results for a follow-up period of 4 or 5 years. At baseline, the marginal bone level change is around -0.30 – -2.24 mm, 1^st^ year of loading -0.85 – -3.70 mm, 2^nd^ year of loading -0.41 – -3.88 mm, 3^rd^ year of loading -1.30 – -4.91 mm, 4^th^ year of loading -1.10 – -4.84 mm, and 5^th^ year of loading -1.57 – -4.76 mm.

### Vertical guided bone regeneration (Table 1, Figure 6)

**Figure 6:**
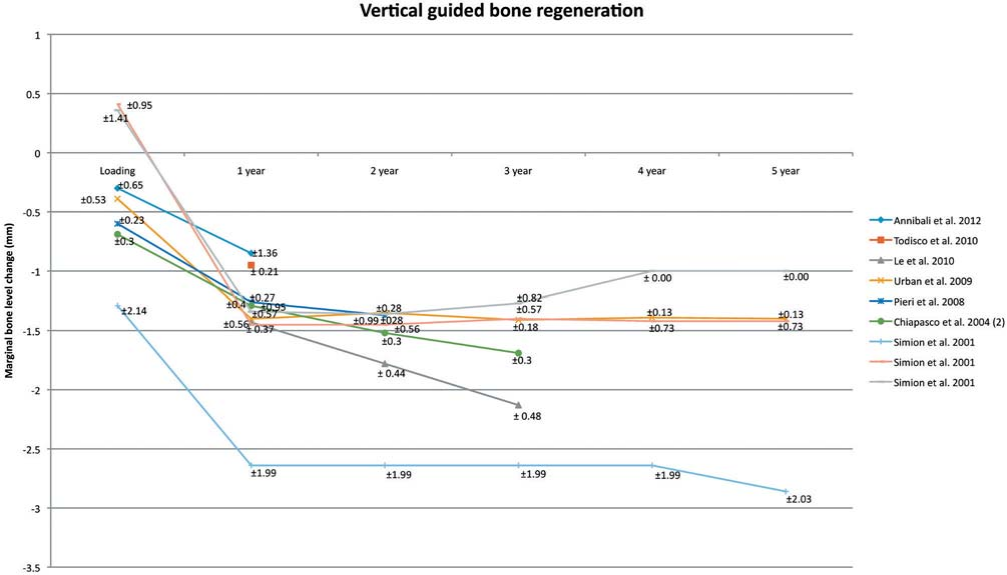
Vertical guided bone regeneration. Mean and Standard Deviation are indicated

The 51 articles included provided seven studies^6^ ^,^ ^22^ ^,^ ^61^ ^,^ ^80^ ^,^ ^96^ ^,^ ^100^ ^,^ ^105^ with vertical bone regeneration. Three studies were retrospective while 4 were prospective. A total of 138 patients with a vertical resorption of partially or totally edentulous alveolar ridges were treated with vertical guided bone regeneration. Two different donor materials for the bone were used: autogenous (ramus^22^ ^,^ ^96^ ^,^ ^105^ and chin^96^ ^,^ ^105^) and allografts^61^ ^,^ ^100^. Moreover, combinations of different donor materials for the bone were used - autogenous+allograft^6^ and autogenous+xenograft^80^. In total, 141 dental implants were placed immediately, 206 dental implants were placed after 4 to 12 months, and the mean was 7.8 months after the healing of the vertical bone regeneration. After the start of loading, the follow-up ranged from 1.0 to 5.3 years, and the mean was 2.4 years. The survival rates for the dental implants in bone from the vertical bone regeneration ranged from 94.1 to 100.0%, and the mean was 99.3%. The implant success rate ranged from 75.0 to 100.0%, and the mean was 90.7%.

All the seven studies^6^ ^,^ ^22^ ^,^ ^61^ ^,^ ^80^ ^,^ ^96^ ^,^ ^100^ ^,^ ^105^ which used vertical bone regeneration as a treatment presented the marginal bone level change in their results. The marginal bone level change is shown in Figure 5. One study^96^ has different treatment groups, therefore, it is shown three times in the figure. Only two studies presented the results for a follow-up period of 5 years. At baseline, the marginal bone level change is around 0.41 – -1.29 mm, 1^st^ year of loading -0.85 – -2.64 mm, 2^nd^ year of loading -1.35 – -2.64 mm, 3^rd^ year of loading -1.27 – -2.64 mm, 4^th^ year of loading -1.00 – -2.64 mm, and 5^th^ year of loading -1.00 – -2.86 mm.

## DISCUSSION

In the literature, evidence is available about the stability of vertical bone augmentation. A wide range of different techniques was used to vertically augment the bone. This review tried to systematically evaluate the current evidence and to compare the different vertical augmentation techniques as well as their marginal bone level change on the long-term. In total, 51 articles could be included, from which the data were obtained. Only 21 articles out of 51 contained information about the marginal bone level change. Line graphs with standard deviation were used to present the marginal bone level change over a long period of time.

Few articles^4^ ^,^ ^5^ ^,^ ^17^ ^,^ ^101^ showing the marginal bone level change around a successful implant are available in literature. In order to assess the stability of an implant in augmented bone, it is important to know the marginal bone level change around a successful implant in non-augmented bone. The most recent data about marginal bone level change around non-augmented implants were discussed at the Third EAO consensus conference. In this article, data of implants in an augmented side were collected and compared with the EAO consensus conference conclusions.

### Alveolar distraction

The analysis shows that the implant survival and success rates are comparable with dental implants which are placed in non-augmented bone^4^. The line graph (Figure 2) shows an overview of the marginal bone level change for the first 5 years. Only three studies present the results for a follow-up period of 4 or 5 years^21^ ^,^ ^57^ ^,^ ^81^. Unfortunately, it was not possible to combine those results. The marginal bone level change between abutment connection and 1^st^ year of loading varies between -0.60 – -0.97 mm. After the 2^nd^ year, it varies between -0.1 – -0.3 mm; after the 3^rd^ year, between -0.06 – -0.17 mm; after the 4^th^ year, between 0 – -0.2 mm; and after the 5^th^ year of loading it is -0.09 mm. These data are in agreement with the present success criteria for the 1^st^ year of loading, which allows a marginal bone loss of 1-1.5 mm^5^ ^,^ ^17^. In the 2^nd^, 3^rd^, 4^th^, and 5^th^ year, the bone loss is, in most of the studies, more than 0.1 mm. This could indicate that the resorption rate is more rapidly progressing compared to non-augmented bone.

Alveolar distraction initiates natural bone formation between the distracted segment and the basal bone. Therefore, there is no need for bone grafting, but for a narrow ridge instead. For a narrow ridge, a bone grafting is better to use, since it can rebuild the horizontal and vertical components. Alveolar distraction seems to be only indicated for the mandible because of the pneumatisation of the sinus in the maxilla. A disadvantage of this technique is the early resorption of the distracted bone. It is essential to consider some overcorrection during treatment planning for directly avoiding surgical relapse and another surgical intervention for additional augmentation. Alveolar distraction undergoes a more active remodeling process because of the better vascularization when compared to a block graft^47^. For the long-term, the marginal bone level change might be more stable.

### Inlay technique

The analysis shows that implant survival and success rates are comparable with dental implants which are placed in non-augmented bone^4^. The line graph (Figure 3) shows an overview of the marginal bone level change for the first 5 years. Only one study^38^ presents a follow-up period of 3 years. Unfortunately, it was not possible to draw any conclusion.

The inlay technique is a technique in which a new graft is placed between the cranial bone segment and the basal bone. The inlay technique in the maxilla is usually seen as a sinus floor augmentation. This part is excluded from this review. For a narrow ridge, a horizontal bone grafting is sometimes needed. A difficulty for the inlay technique is the management of soft tissues. The soft tissues need to maintain sufficient blood supply to the bone segment which is cranially displaced. The risk of wound dehiscence could arise when there is too much tension after wound closure. Unfortunately, no long-term follow-up studies are available. Therefore, a comparison with dental implants in non-augmented bone is not possible.

### Onlay technique

The analysis shows that implant survival and success rates are comparable with dental implants which are placed in non-augmented bone. The line graph (Figure 4) shows an overview of the marginal bone level change for the first 5 years. Only four studies present the results for a follow-up period of 4 or 5 years^25^ ^,^ ^29^ ^,^ ^57^ ^,^ ^74^. Unfortunately, it was not possible to combine those results. The marginal bone level change between abutment connection and 1^st^ year of loading varies between -0.60 – -1.46 mm; after the 2^nd^ year, between -0.03 – 0.30 mm; after the 3^rd^ year, between -0.03 – -1.03 mm; after the 4^th^ year, between 0.2 – -0.06 mm; and after the 5^th^ year of loading, between 0.08 – -0.27 mm. These data are in agreement with the present success criteria for the 1^st^ year of loading, which allows a marginal bone loss of 1-1.5 mm, and of 0.1 mm for the 2^nd^, 3^rd^, 4^th^, and 5^th^ year^5^ ^,^ ^17^. However, one study^74^ showed more marginal bone loss in comparison with others^25^ ^,^ ^29^ ^,^ ^57^.

The onlay technique is done mostly with an autogenous bone graft. Before the year 2000, most implants were immediately placed together with the bone grafts. The implants were used to secure the graft. The capacity and volume of the bone grafts are variable between the studies. These differences could be explained by different follow-up periods, timing of implants placement, different sites, and different bone grafting material. Overall, the resorption rate is higher in the first year, but stabilizes after it. The autogenous bone graft is still the most frequently used graft for the onlay technique. It is a recommendation to use corticocancellous bone instead of particulated bone grafts. Ideally, oversized grafts should be harvested to maintain enough volume after the initial resorption phase. The major difficulty for the onlay technique is the management of the soft tissues to maintain a full wound closure. For the long-term, it seems that the marginal bone level change is comparable with dental implants in non-augmented bone.

### Vertical guided bone regeneration

The analysis shows that the implant survival is comparable whereas the success rate is not comparable with dental implants which are placed in non-augmented bone. The line graph (Figure 5) shows an overview of the marginal bone level change for the first 5 years. Only two studies present the results for a follow-up period of 5 years^96^ ^,^ ^105^. Unfortunately, once again it is not possible to combine those results. The marginal bone level change between abutment connection and 1^st^ year of loading varies between -1.01 – -1.86 mm; after the 2^nd^ year, between 0.05 – -0.02 mm; after the 3^rd^ year, between 0.11 – -0.06 mm; after the 4^th^ year, between 0.27 – -0.02 mm; and after the 5^th^ year of loading, between 0 – -0.22 mm. These data are in agreement with the present success criteria for the 1^st^ year of loading, which allows a marginal bone loss during the first year of 1-1.5 mm, and of 0.1 mm for the 2^nd^, 3^rd^, 4^th^, and 5^th^ year^5^ ^,^ ^17^. However, one study^96^ has a different amount of dental implants during the follow-up period, which could influence the outcome.

Vertical guided bone regeneration implies that the regeneration of osseous defects is predictably attainable via the application of occlusive membranes, which mechanically exclude non-osteogenic cell populations from the surrounding soft tissues. In the past, non-resorbable membranes were used, but nowadays resorbable membranes are common. The defect is always filled with particulate autogenous bone, and sometimes mixed with xenograft or allograft. Wound dehiscence is often seen as a complication. Therefore, it is important to get as little traction on the wound as possible. For the long-term, it seems that the marginal bone level loss is comparable with dental implants in non augmented bone.

In the literature, a lot of different criteria is used to determine the survival and success rates of dental implants. The lack of universally accepted success criteria makes the interpretation and comparison of the data really difficult^76^. In addition, a statistical problem is perceived. There is a discrepancy in reported outcomes when the primary unit of analysis is the patient instead of the dental implant^87^ ^,^ ^88^. Therefore, the decision is made to show all the data which criterion or statistical analysis has been used. This could be a disadvantage, but it gives the clinician a complete overview of the available literature.

Some new guidelines were proposed in the VIII European Workshop on Periodontology. A successful dental implant has to meet criteria concerning tissue physiology (osseointegration), function (chewing), absence of pain, and user satisfaction^101^. The first criteria for marginal bone loss exist since 1986^5^. This review shows that the marginal bone loss after abutment connection and the first year of loading varies between 1.0 and 1.5 mm. This is called saucerisation, and is caused by the establishment of the biological width. Recent studies allow a mean marginal bone loss of 1.0 mm in the first year of loading, and an annual of 0.1 mm bone loss can be expected in the following years ^17^. The criteria are divided into three domains that are important for identifying the success of a dental implant. These domains are: patient-reported outcome measures (health-related quality of live and general satisfaction), peri-implant health (marginal bone level, bleeding on probing, and probing depth), and implant-supported restorations (longevity of the restoration, function/occlusion related outcomes, and technical complications)^101^.

To give a complete overview about the different techniques, every type of grafting material was included. Depending on the grafting material used, a different resorption occurs. That is why the results are presented in graphs and tables, which facilitates the decision of clinicians regarding what type of grafting material must be used. No distinction is made between the different durations of the follow-up period, even though there was a wide range of it. The follow-up period needs to be of at least one year. These different lengths of follow-up periods are included in the calculations. However, an implant success rate of 100% after one year cannot be compared with a success rate after 10 years. Furthermore, different follow-up periods per patient in a study are pooled together. This could lead to a complete different outcome. This review is designed to give a complete overview, thus, the clinician can decide what the best treatment is.

After analysis of the articles about vertical bone augmentation, the main conclusion was that a wide range of different techniques and materials were used, and also different patient groups, study designs, antibiotic prescriptions, and follow-up regimes. Because of this, no meta-analysis was conducted, for once a meta-analysis is performed, it causes a bias.

Another limitation of this review is that it was not possible to separate the data for single tooth gap, multiple missing teeth, or an edentulous ridge in the different articles used. These different clinical situations were mostly pooled together; therefore, it was hard to analyze a specific technique for a specific clinical condition. For most defect and especially in the atrophic jaws, the description of the seize of the defect was hardly present, which was also a topic in the last ITI Consensus Conference^11^.

Based on our previous findings, it is hard to state which vertical bone augmentation is the best to use. However, when only considering those vertical bone augmentation techniques for which studies exist with a follow-up period of at least 4 to 5 years, there seems to be a trend that the onlay technique, alveolar distraction, and vertical guided bone regeneration are stable for at least 4 to 5 years. Since it was not possible to carry out meta-analytic procedures, a conclusion about stability is not justified, but a trend is still visible. However, further research is necessary to clarify this finding. More studies that follow the marginal bone level change for a longer period are necessary, in addition to better description and ridge measurements of the clinical situation before and after the augmentation procedure. This will enable a better interpretation of the results and allow the clinician to conclude which specific augmentation is recommended and in which clinical situation.

## References

[1] Acocella A, Bertolai R, Ellis E, Nissan J, Sacco R (2012). Maxillary alveolar ridge reconstruction with monocortical fresh-frozen bone blocks: a clinical, histological and histomorphometric study. J Craniomaxillofac Surg.

[2] Adell R, Lekholm U, Gröndahl K, Brånemark PI, Lindström J, Jacobsson M (1990). Reconstruction of severely resorbed edentulous maxillae using osseointegrated fixtures in immediate autogenous bone grafts. Int J Oral Maxillofac Implants.

[3] Adell R, Lekholm U, Rockler B, Brånemark PI (1981). A 15-year study of osseointegrated implants in the treatment of the edentulous jaw. Int J Oral Surg.

[4] Albrektsson T, Donos N (2012). Working Group 1. Implant survival and complications. The Third EAO consensus conference 2012. Clin Oral Implants Res.

[5] Albrektsson T, Zarb G, Worthington P, Eriksson AR (1986). The long-term efficacy of currently used dental implants: a review and proposed criteria of success. Int J Oral Maxillofac Implants.

[6] Annibali S, Bignozzi I, Sammartino G, La Monaca G, Cristalli MP (2012). Horizontal and vertical ridge augmentation in localized alveolar deficient sites: a retrospective case series. Implant Dent.

[7] Astrand P, Nord PG, Branemark PI (1996). Titanium implants and onlay bone graft to the atrophic edentulous maxilla: a 3-year longitudinal study. Int J Oral Maxillofac Surg.

[8] Becktor JP, Isaksson S, Sennerby L (2004). Survival analysis of endosseous implants in grafted and nongrafted edentulous maxillae. Int J Oral Maxillofac Implants.

[9] Bell RB, Blakey GH, White RP, Hillebrand DG, Molina A (2002). Staged reconstruction of the severely atrophic mandible with autogenous bone graft and endosteal implants. J Oral Maxillofac Surg.

[10] Bianchi A, Felice P, Lizio G, Marchetti C (2008). Alveolar distraction osteogenesis versus inlay bone grafting in posterior mandibular atrophy: a prospective study. Oral Surg Oral Med Oral Pathol Oral Radiol Endod.

[11] Bornstein MM, Al-Nawas B, Kuchler U, Tahmaseb A (2014). Consensus statements and recommended clinical procedures regarding contemporary surgical and radiographic techniques in implant dentistry. Int J Oral Maxillofac Implants.

[12] Bravi F, Bruschi GB, Ferrini F (2007). A 10-year multicenter retrospective clinical study of 1715 implants placed with the edentulous ridge expansion technique. Int J Periodontics Restorative Dent.

[13] Browaeys H, Bouvry P, De Bruyn H (2007). A literature review on biomaterials in sinus augmentation procedures. Clin Implant Dent Relat Res.

[14] Buser D, Ingimarsson S, Dula K, Lussi A, Hirt HP, Belser UC (2002). Long-term stability of osseointegrated implants in augmented bone: a 5-year prospective study in partially edentulous patients. Int J Periodontics Restorative Dent.

[15] Canullo L, Sisti A (2010). Early implant loading after vertical ridge augmentation (VRA) using e-PTFE titanium-reinforced membrane and nano-structured hydroxyapatite: 2-year prospective study. Eur J Oral Implantol.

[16] Carinci F, Brunelli G, Zollino I, Franco M, Viscioni A, Rigo L (2009). Mandibles grafted with fresh-frozen bone: an evaluation of implant outcome. Implant Dent.

[17] Cecchinato D, Bengazi F, Blasi G, Botticelli D, Cardarelli I, Gualini F (2008). Bone level alterations at implants placed in the posterior segments of the dentition: outcome of submerged/non-submerged healing. A 5-year multicenter, randomized, controlled clinical trial. Clin Oral Implants Res.

[18] Chiapasco M, Abati S, Romeo E, Vogel G (1999). Clinical outcome of autogenous bone blocks or guided bone regeneration with e-PTFE membranes for the reconstruction of narrow edentulous ridges. Clin Oral Implants Res.

[19] Chiapasco M, Casentini P, Zaniboni M (2009). Bone augmentation procedures in implant dentistry. Int J Oral Maxillofac Implants.

[20] Chiapasco M, Casentini P, Zaniboni M, Corsi E (2012). Evaluation of peri-implant bone resorption around Straumann Bone Level implants placed in areas reconstructed with autogenous vertical onlay bone grafts. Clin Oral Implants Res.

[21] Chiapasco M, Consolo U, Bianchi A, Ronchi P (2004). Alveolar distraction osteogenesis for the correction of vertically deficient edentulous ridges: a multicenter prospective study on humans. Int J Oral Maxillofac Implants.

[22] Chiapasco M, Romeo E, Casentini P, Rimondini L (2004). Alveolar distraction osteogenesis vs. vertical guided bone regeneration for the correction of vertically deficient edentulous ridges: a 1-3-year prospective study on humans. Clin Oral Implants Res.

[23] Chiapasco M, Romeo E, Coggiola A, Brusati R (2011). Long-term outcome of dental implants placed in revascularized fibula free flaps used for the reconstruction of maxillo-mandibular defects due to extreme atrophy. Clin Oral Implants Res.

[24] Chiapasco M, Zaniboni M, Boisco M (2006). Augmentation procedures for the rehabilitation of deficient edentulous ridges with oral implants. Clin Oral Implants Res.

[25] Chiapasco M, Zaniboni M, Rimondini L (2007). Autogenous onlay bone grafts vs. alveolar distraction osteogenesis for the correction of vertically deficient edentulous ridges: a 2-4-year prospective study on humans. Clin Oral Implants Res.

[26] Chin M (1999). Distraction osteogenesis for dental implants. Atlas Oral Maxillofac Surg Clin North Am.

[27] Cordaro L, Torsello F, Accorsi Ribeiro C, Liberatore M, Mirisola di Torresanto V (2010). Inlay-onlay grafting for three-dimensional reconstruction of the posterior atrophic maxilla with mandibular bone. Int J Oral Maxillofac Surg.

[28] Cordaro L, Torsello F, Miuccio MT, di Torresanto VM, Eliopoulos D (2011). Mandibular bone harvesting for alveolar reconstruction and implant placement: subjective and objective cross-sectional evaluation of donor and recipient site up to 4 years. Clin Oral Implants Res.

[29] Corinaldesi G, Pieri F, Sapigni L, Marchetti C (2009). Evaluation of survival and success rates of dental implants placed at the time of or after alveolar ridge augmentation with an autogenous mandibular bone graft and titanium mesh: a 3- to 8-year retrospective study. Int J Oral Maxillofac Implants.

[30] Dahlin C, Johansson A (2011). Iliac crest autogenous bone graft versus alloplastic graft and guided bone regeneration in the reconstruction of atrophic maxillae: a 5-year retrospective study on cost-effectiveness and clinical outcome. Clin Implant Dent Relat Res.

[31] De Riu G, Meloni MS, Pisano M, Baj A, Tullio A (2012). Mandibular coronoid process grafting for alveolar ridge defects. Oral Surg Oral Med Oral Pathol Oral Radiol.

[32] Den Hartog L, Slater JJ, Vissink A, Meijer HJ, Raghoebar GM (2008). Treatment outcome of immediate, early and conventional single-tooth implants in the aesthetic zone: a systematic review to survival, bone level, soft-tissue, aesthetics and patient satisfaction. J Clin Periodontol.

[33] Dinopoulos H, Dimitriou R, Giannoudis PV (2012). Bone graft substitutes: What are the options?. Surgeon.

[34] Donovan MG, Dickerson NC, Hanson LJ, Gustafson RB (1994). Maxillary and mandibular reconstruction using calvarial bone grafts and Branemark implants: a preliminary report. J Oral Maxillofac Surg.

[35] Elian N, Jalbout Z, Ehrlich B, Classi A, Cho SC, Al-Kahtani F (2008). A two-stage full-arch ridge expansion technique: review of the literature and clinical guidelines. Implant Dent.

[36] Elo JA, Herford AS, Boyne PJ (2009). Implant success in distracted bone versus autogenous bone-grafted sites. J Oral Implantol.

[37] Enislidis G, Fock N, Millesi-Schobel G, Klug C, Wittwer G, Yerit K (2005). Analysis of complications following alveolar distraction osteogenesis and implant placement in the partially edentulous mandible. Oral Surg Oral Med Oral Pathol Oral Radiol Endod.

[38] Esposito M, Cannizarro G, Soardi E, Pellegrino G, Pistilli R, Felice P (2011). A 3-year post-loading report of a randomised controlled trial on the rehabilitation of posterior atrophic mandibles: short implants or longer implants in vertically augmented bone?. Eur J Oral Implantol.

[39] Esposito M, Pellegrino G, Pistilli R, Felice P (2011). Rehabilitation of posterior atrophic edentulous jaws: prostheses supported by 5 mm short implants or by longer implants in augmented bone? One-year results from a pilot randomised clinical trial. Eur J Oral Implantol.

[40] Ettl T, Gerlach T, Schüsselbauer T, Gosau M, Reichert TE, Driemel O (2010). Bone resorption and complications in alveolar distraction osteogenesis. Clin Oral Investig.

[41] Felice P, Pellegrino G, Checchi L, Pistilli R, Esposito M (2010). Vertical augmentation with interpositional blocks of anorganic bovine bone vs. 7-mm-long implants in posterior mandibles: 1-year results of a randomized clinical trial. Clin Oral Implants Res.

[42] Felice P, Pistilli R, Lizio G, Pellegrino G, Nisii A, Marchetti C (2009). Inlay versus onlay iliac bone grafting in atrophic posterior mandible: a prospective controlled clinical trial for the comparison of two techniques. Clin Implant Dent Relat Res.

[43] Gaggl A, Schultes G, Kärcher H (2000). Vertical alveolar ridge distraction with prosthetic treatable distractors: a clinical investigation. Int J Oral Maxillofac Implants.

[44] Greenberg JA, Wiltz MJ, Kraut RA (2012). Augmentation of the anterior maxilla with intraoral onlay grafts for implant placement. Implant Dent.

[45] Gutta R, Waite PD (2009). Outcomes of calvarial bone grafting for alveolar ridge reconstruction. Int J Oral Maxillofac Implants.

[46] Higgins JPT, Green S (2011). Cochrane Handbook for Systematic Reviews of Interventions Version 5.1.0 [updated March 2011].

[47] Hodges NE, Perry M, Mohamed W, Hallmon WW, Rees T, Opperman LA (2006). Distraction osteogenesis versus autogenous onlay grafting. Part II: biology of regenerate and onlay bone. Int J Oral Maxillofac Implants.

[48] Jensen J, Sindet-Pedersen S (1991). Autogenous mandibular bone grafts and osseointegrated implants for reconstruction of the severely atrophied maxilla: a preliminary report. J Oral Maxillofac Surg.

[49] Jensen OT (2006). Alveolar segmental "sandwich" osteotomies for posterior edentulous mandibular sites for dental implants. J Oral Maxillofac Surg.

[50] Jensen OT, Cockrell R, Kuhike L, Reed C (2002). Anterior maxillary alveolar distraction osteogenesis: a prospective 5-year clinical study. Int J Oral Maxillofac Implants.

[51] Jensen OT, Kuhlke L, Bedard JF, White D (2006). Alveolar segmental sandwich osteotomy for anterior maxillary vertical augmentation prior to implant placement. J Oral Maxillofac Surg.

[52] Kahnberg KE, Nystrom E, Bartholdsson L (1989). Combined use of bone grafts and Brånemark fixtures in the treatment of severely resorbed maxillae. Int J Oral Maxillofac Implants.

[53] Kawakami PY, Dottore AM, Bechara K, Feres M, Shibli JA (2013). Alveolar osteotomy associated with resorbable non-ceramic hydroxylapatite or intra-oral autogenous bone for height augmentation in posterior mandibular sites: a split-mouth prospective study. Clin Oral Implants Res.

[54] Keller EE, Eckert SE, Tolman DE (1994). Maxillary antral and nasal one-stage inlay composite bone graft: preliminary report on 30 recipient sites. J Oral Maxillofac Surg.

[55] Keller EE, Tolman DE, Eckert S (1999). Surgical-prosthodontic reconstruction of advanced maxillary bone compromise with autogenous onlay block bone grafts and osseointegrated endosseous implants: a 12-year study of 32 consecutive patients. Int J Oral Maxillofac Implants.

[56] Khojasteh A, Behnia H, Shayesteh YS, Morad G, Alikhasi M (2012). Localized bone augmentation with cortical bone blocks tented over different particulate bone substitutes: a retrospective study. Int J Oral Maxillofac Implants.

[57] Kim JW, Cho MH, Kim SJ, Kim MR (2013). Alveolar distraction osteogenesis versus autogenous onlay bone graft for vertical augmentation of severely atrophied alveolar ridges after 12 years of long-term follow-up. Oral Surg Oral Med Oral Pathol Oral Radiol.

[58] Korpi JT, Kainulainen VT, Sandor GK, Oikarinen KS (2012). Long-term follow-up of severely resorbed mandibles reconstructed using tent pole technique without platelet-rich plasma. J Oral Maxillofac Surg.

[59] Laverick S, Summerwill A, Cawood JI (2008). Ten years of experience with the anterior maxillary and mandibular osteoplasty (class IV ridges): a retrospective analysis of implant survival rates. Int J Oral Maxillofac Surg.

[60] Le B, Burstein J, Sedghizadeh PP (2008). Cortical tenting grafting technique in the severely atrophic alveolar ridge for implant site preparation. Implant Dent.

[61] Le B, Rohrer MD, Prasad HS (2010). Screw "tent-pole" grafting technique for reconstruction of large vertical alveolar ridge defects using human mineralized allograft for implant site preparation. J Oral Maxillofac Surg.

[62] Lekholm U, Wannfors K, Isaksson S, Adielsson B (1999). Oral implants in combination with bone grafts. A 3-year retrospective multicenter study using the Brånemark implant system. Int J Oral Maxillofac Surg.

[63] Levin L, Nitzan D, Schwartz-Arad D (2007). Success of dental implants placed in intraoral block bone grafts. J Periodontol.

[64] Li L, Blake F, Heiland M, Schmelzle R, Pohlenz P (2007). Long-term evaluation after mandibular reconstruction with fibular grafts versus microsurgical fibular flaps. J Oral Maxillofac Surg.

[65] Ludovichetti M, Di Stefano DA, Pagnutti S, Vaccari E, Ludovichetti FS, Celletti R (2011). Vertical ridge augmentation using a flexible heterologous cortical bone sheet: three-year follow-up. Int J Periodontics Restorative Dent.

[66] McGrath CJ, Schepers SH, Blijdorp PA, Hoppenreijs TJ, Erbe M (1996). Simultaneous placement of endosteal implants and mandibular onlay grafting for treatment of the atrophic mandible. A preliminary report. Int J Oral Maxillofac Surg.

[67] Milinkovic I, Cordaro L (2014). Are there specific indications for the different alveolar bone augmentation procedures for implant placement? A systematic review. Int J Oral Maxillofac Implants.

[68] Miyamoto I, Funaki K, Yamauchi K, Kodama T, Takahashi T (2012). Alveolar ridge reconstruction with titanium mesh and autogenous particulate bone graft: computed tomography-based evaluations of augmented bone quality and quantity. Clin Implant Dent Relat Res.

[69] Moher D, Liberati A, Tetzlaff J, Altman DG, PRISMA Group (2009). Preferred reporting items for systematic reviews and meta-analyses: the PRISMA statement. J Clin Epidemiol.

[70] Nissan J, Ghelfan O, Mardinger O, Calderon S, Chaushu G (2011). Efficacy of cancellous block allograft augmentation prior to implant placement in the posterior atrophic mandible. Clin Implant Dent Relat Res.

[71] Nissan J, Mardinger O, Calderon S, Romanos GE, Chaushu G (2011). Cancellous bone block allografts for the augmentation of the anterior atrophic maxilla. Clin Implant Dent Relat Res.

[72] Nissan J, Marilena V, Gross O, Mardinger O, Chaushu G (2012). Histomorphometric analysis following augmentation of the anterior atrophic maxilla with cancellous bone block allograft. Int J Oral Maxillofac Implants.

[73] Novell J, Novell-Costa F, Ivorra C, Fariñas O, Munilla A, Martinez C (2012). Five-year results of implants inserted into freeze-dried block allografts. Implant Dent.

[74] Nyström E, Ahlqvist J, Gunne J, Kahnberg KE (2004). 10-year follow-up of onlay bone grafts and implants in severely resorbed maxillae. Int J Oral Maxillofac Surg.

[75] Nyström E, Ahlqvist J, Legrell PE, Kahnberg KE (2002). Bone graft remodelling and implant success rate in the treatment of the severely resorbed maxilla: a 5-year longitudinal study. Int J Oral Maxillofac Surg.

[76] Ong CT, Ivanovski S, Needleman IG, Retzepi M, Moles DR, Tonetti MS (2008). Systematic review of implant outcomes in treated periodontitis subjects. J Clin Periodontol.

[77] Palmer P, Palmer R (1999). Dental implants. 8. Implant surgery to overcome anatomical difficulties. Br Dent J.

[78] Pelo S, Boniello R, Moro A, Gasparini G, Amoroso PF (2010). Augmentation of the atrophic edentulous mandible by a bilateral two-step osteotomy with autogenous bone graft to place osseointegrated dental implants. Int J Oral Maxillofac Surg.

[79] Pérez-Sayáns M, León-Camacho ML, Somoza-Martín JM, Fernández-González B, Blanes-Vázquez-Gundín S, Gándara-Rey JM (2013). Dental implants placed on bone subjected to vertical alveolar distraction show the same performance as those placed on primitive bone. Med Oral Patol Oral Cir Bucal.

[80] Pieri F, Corinaldesi G, Fini M, Aldini NN, Giardino R, Marchetti C (2008). Alveolar ridge augmentation with titanium mesh and a combination of autogenous bone and anorganic bovine bone: a 2-year prospective study. J Periodontol.

[81] Polo WC, Araujo NS, Lima YB, Joly JC, Sendyk WR, Cury PR (2007). Peri-implant bone loss around posterior mandible dental implants placed after distraction osteogenesis: preliminary findings. J Periodontol.

[82] Rachmiel A, Srouji S, Peled M (2001). Alveolar ridge augmentation by distraction osteogenesis. Int J Oral Maxillofac Surg.

[83] Raghoebar GM, Liem RS, Vissink A (2002). Vertical distraction of the severely resorbed edentulous mandible: a clinical, histological and electron microscopic study of 10 treated cases. Clin Oral Implants Res.

[84] Raghoebar GM, Schoen P, Meijer HJ, Stellingsma K, Vissink A (2003). Early loading of endosseous implants in the augmented maxilla: a 1-year prospective study. Clin Oral Implants Res.

[85] Rigo L, Viscioni A, Franco M, Lucchese A, Zollino I, Brunelli G (2011). Overdentures on implants placed in bone augmented with fresh frozen bone. Minerva Stomatol.

[86] Robiony M, Zorzan E, Polini F, Sembronio S, Toro C, Politi M (2008). Osteogenesis distraction and platelet-rich plasma: combined use in restoration of severe atrophic mandible. Long-term results. Clin Oral Implants Res.

[87] Roos-Jansåker AM, Lindahl C, Renvert H, Renvert S (2006). Nine- to fourteen-year follow-up of implant treatment. Part I: implant loss and associations to various factors. J Clin Periodontol.

[88] Roos-Jansåker AM, Renvert H, Lindahl C, Renvert S (2006). Nine- to fourteen-year follow-up of implant treatment. Part III: factors associated with peri-implant lesions. J Clin Periodontol.

[89] Satow S, Slagter AP, Stoelinga PJ, Habets LL (1997). Interposed bone grafts to accommodate endosteal implants for retaining mandibular overdentures. A 1-7 year follow-up study. Int J Oral Maxillofac Surg.

[90] Sbordone C, Toti P, Guidetti F, Califano L, Santoro A, Sbordone L (2012). Volume changes of iliac crest autogenous bone grafts after vertical and horizontal alveolar ridge augmentation of atrophic maxillas and mandibles: a 6-year computerized tomographic follow-up. J Oral Maxillofac Surg.

[91] Sbordone L, Toti P, Menchini-Fabris G, Sbordone C, Guidetti F (2009). Implant survival in maxillary and mandibular osseous onlay grafts and native bone: a 3-year clinical and computerized tomographic follow-up. Int J Oral Maxillofac Implants.

[92] Sbordone L, Toti P, Menchini-Fabris GB, Sbordone C, Piombino P, Guidetti F (2009). Volume changes of autogenous bone grafts after alveolar ridge augmentation of atrophic maxillae and mandibles. Int J Oral Maxillofac Surg.

[93] Schliephake H, Neukam FW, Wichmann M (1997). Survival analysis of endosseous implants in bone grafts used for the treatment of severe alveolar ridge atrophy. J Oral Maxillofac Surg.

[94] Sethi A, Kaus T (2001). Ridge augmentation using mandibular block bone grafts: preliminary results of an ongoing prospective study. Int J Oral Maxillofac Implants.

[95] Sezer B, Koyuncu BO, Gunbay T, Sezak M (2012). Alveolar distraction osteogenesis in the human mandible: a clinical and histomorphometric study. Implant Dent.

[96] Simion M, Jovanovic SA, Tinti C, Benfenati SP (2001). Long-term evaluation of osseointegrated implants inserted at the time or after vertical ridge augmentation. A retrospective study on 123 implants with 1-5 year follow-up. Clin Oral Implants Res.

[97] Simonis P, Dufour T, Tenenbaum H (2010). Long-term implant survival and success: a 10-16-year follow-up of non-submerged dental implants. Clin Oral Implants Res.

[98] Sjöström M, Sennerby L, Nilson H, Lundgren S (2007). Reconstruction of the atrophic edentulous maxilla with free iliac crest grafts and implants: a 3-year report of a prospective clinical study. Clin Implant Dent Relat Res.

[99] Smolka W, Bosshardt DD, Mericske-Stern R, Iizuka T (2006). Reconstruction of the severely atrophic mandible using calvarial split bone grafts for implant-supported oral rehabilitation. Oral Surg Oral Med Oral Pathol Oral Radiol Endod.

[100] Todisco M (2010). Early loading of implants in vertically augmented bone with non-resorbable membranes and deproteinised anorganic bovine bone. An uncontrolled prospective cohort study. Eur J Oral Implantol.

[101] Tonetti M, Palmer R, Working Group 2 of the VEWoP (2012). Clinical research in implant dentistry: study design, reporting and outcome measurements: consensus report of Working Group 2 of the VIII European Workshop on Periodontology. J Clin Periodontol.

[102] Torres J, Tamimi F, Alkhraisat MH, Manchón A, Linares R, Prados-Frutos JC (2010). Platelet-rich plasma may prevent titanium-mesh exposure in alveolar ridge augmentation with anorganic bovine bone. J Clin Periodontol.

[103] Triplett RG, Schow SR (1996). Autologous bone grafts and endosseous implants: complementary techniques. J Oral Maxillofac Surg.

[104] Uckan S, Oguz Y, Bayram B (2007). Comparison of intraosseous and extraosseous alveolar distraction osteogenesis. J Oral Maxillofac Surg.

[105] Urban IA, Jovanovic SA, Lozada JL (2009). Vertical ridge augmentation using guided bone regeneration (GBR) in three clinical scenarios prior to implant placement: a retrospective study of 35 patients 12 to 72 months after loading. Int J Oral Maxillofac Implants.

[106] Van der Meij EH, Blankestijn J, Berns RM, Bun RJ, Jovanovic A, Onland JM (2005). The combined use of two endosteal implants and iliac crest onlay grafts in the severely atrophic mandible by a modified surgical approach. Int J Oral Maxillofac Surg.

[107] Verdugo F, Simonian K, Frydman A, D'Addona A, Pontón J (2011). Long-term block graft stability in thin periodontal biotype patients: a clinical and tomographic study. Int J Oral Maxillofac Implants.

[108] Verhoeven JW, Cune MS, Ruijter J (2006). Permucosal implants combined with iliac crest onlay grafts used in extreme atrophy of the mandible: long-term results of a prospective study. Clin Oral Implants Res.

[109] Verhoeven JW, Cune MS, Terlou M, Zoon MA, de Putter C (1997). The combined use of endosteal implants and iliac crest onlay grafts in the severely atrophic mandible: a longitudinal study. Int J Oral Maxillofac Surg.

[110] Vermeeren JI, Wismeijer D, van Waas MA (1996). One-step reconstruction of the severely resorbed mandible with onlay bone grafts and endosteal implants. A 5-year follow-up. Int J Oral Maxillofac Surg.

[111] Von Arx T, Wallkamm B, Hardt N (1998). Localized ridge augmentation using a micro titanium mesh: a report on 27 implants followed from 1 to 3 years after functional loading. Clin Oral Implants Res.

[112] Wheeler SL (1997). Sinus augmentation for dental implants: the use of alloplastic materials. J Oral Maxillofac Surg.

[113] Widmark G, Andersson B, Andrup B, Carlsson GE, Ivanoff CJ, Lindvall AM (1998). Rehabilitation of patients with severely resorbed maxillae by means of implants with or without bone grafts. A 1-year follow-up study. Int J Oral Maxillofac Implants.

[114] Zwetyenga N, Vidal N, Ella B, Siberchicot F, Emparanza A (2012). Results of oral implant-supported prostheses after mandibular vertical alveolar ridge distraction: a propos of 54 sites. Oral Surg Oral Med Oral Pathol Oral Radiol.

